# Characterization of fast-decaying PET radiotracers solely through LC-MS/MS of constituent radioactive and carrier isotopologues

**DOI:** 10.1186/2191-219X-3-3

**Published:** 2013-01-12

**Authors:** H Umesha Shetty, Cheryl L Morse, Yi Zhang, Victor W Pike

**Affiliations:** 1 Molecular Imaging Branch, National Institute of Mental Health, National Institutes of Health, Building 10, Room B3 C351, 10 Center Drive, MSC 1003, Bethesda, MD, 20892-1003, USA

**Keywords:** PET radiotracers, Carbon-11, Specific radioactivity, LC-MS/MS

## Abstract

**Background:**

The characterization of fast-decaying radiotracers that are labeled with carbon-11 (*t*_1/2_ = 20.38 min), including critical measurement of specific radioactivity (activity per mole at a specific time) before release for use in positron-emission tomography (PET), has relied heavily on chromatographic plus radiometric measurements, each of which may be vulnerable to significant errors. Thus, we aimed to develop a mass-specific detection method using sensitive liquid chromatography-mass spectrometry/mass spectrometry (LC-MS/MS) for identifying ^11^C-labeled tracers and for verifying their specific radioactivities.

**Methods:**

The LC-MS/MS was tuned and set up with methods to generate and measure the product ions specific for carbon-11 species and M + 1 carrier (predominantly the carbon-13 isotopologue) in four ^11^C-labeled tracers. These radiotracers were synthesized and then analyzed before extensive carbon-11 decay. The peak areas of carbon-11 species and M + 1 carrier from the LC-MS/MS measurement and the calculated abundances of carbon-12 carrier and M + 1 radioactive species gave the mole fraction of carbon-11 species in each sample. This value upon multiplication with the theoretical specific radioactivity of carbon-11 gave the specific radioactivity of the radiotracer.

**Results:**

LC-MS/MS of each ^11^C-labeled tracer generated the product ion peaks for carbon-11 species and M + 1 carrier at the expected LC retention time. The intensity of the radioactive peak diminished as time elapsed and was undetectable after six half-lives of carbon-11. Measurements of radiotracer-specific radioactivity determined solely by LC-MS/MS at timed intervals gave a half-life for carbon-11 (20.43 min) in excellent agreement with the value obtained radiometrically. Additionally, the LC-MS/MS measurement gave specific radioactivity values (83 to 505 GBq/μmol) in good agreement with those from conventional radiometric methods.

**Conclusions:**

^11^C-Labeled tracers were characterized at a fundamental level involving isolation and mass detection of extremely low-abundance carbon-11 species along with the M + 1 carrier counterpart. This LC-MS/MS method for characterizing fast-decaying radiotracers is valuable in both the development and production of PET radiopharmaceuticals.

## Background

Positron-emission tomography (PET), as an expanding biomedical research and diagnostic imaging technique, relies on the use of radiotracers labeled with a positron emitter, often either carbon-11 (*t*_1/2_ = 20.38 min) or fluorine-18 (*t*_1/2_ = 109.7 min) [1-3]. The short physical half-lives of these radiotracers demand rapid techniques for establishing the quality of each production batch before release. A suitable technique for this purpose must conclusively identify the radiotracer and also provide specific radioactivity (SA), the ratio of the amount of radioactive compound (GBq) to the amount of compound (mol) in radioactive and non-radioactive forms (isotopologues) at a specific measurement time. High-performance liquid chromatography (HPLC) is almost universally applied to assure radiotracer identity and to measure radiochemical purity and SA at the end of each radiotracer synthesis [4,5]. Typically, ^11^C-labeled tracers are produced with only a low degree of ^11^C enrichment (<0.1%) [1], representing a SA value on the order of 100 GBq/μmol. Nevertheless, an accurate determination of SA is critical in many applications especially where the radiotracer is intended to bind to low-density protein targets, such as neurotransmitter receptors, enzymes or transporters, or in specific cases where the radiotracer is pharmacologically very potent, such as some radiotracers for dopamine, nicotinic, or opiate receptors [6-8].

The identification of a PET radiotracer with HPLC relies on comparison of its retention time (*t*_R_) measured with a radioactivity detector with that of the reference non-radioactive compound measured with another detector, often an absorbance detector. In conventional measurement of the SA of a radiotracer, the detector giving the mass response is calibrated for compound mass, and the radioactivity associated with a particular carrier mass peak is measured in a calibrated detector, invariably a dose calibrator (an ionization detector). Surrogate radioisotopes (e.g., ^137^Cs, ^57^Co) are often used to calibrate ionization detectors for measuring positron emitters. The accuracy of measurements is highly vulnerable to errors in instrument settings, and to changes in sample containers and geometries, as demonstrated in several studies with fluorine-18 [9-14]. Similar issues will pertain to shorter-lived carbon-11, although this has not been well studied [15]. In addition, the presence of unknown inadvertent impurities in the radiotracer may compromise the mass measurements and hence the accuracy of derived SA. Hitherto, no non-radiometric method for verifying conventional HPLC-radiometric measurements of ^11^C-labeled tracer SA has been reported.

We recognized the potential of a highly sensitive mass spectrometric technique for developing a reliable method to characterize fast-decaying PET radiotracers. Such a method seemed practical using triple quadrupole liquid chromatography-mass spectrometry/mass spectrometry (LC-MS/MS) because its specificity, sensitivity, and detection range were envisaged to be adequate for measuring both the radioactive species and the carrier counterpart. Additionally, the MS/MS can generate, isolate, and measure ions specifically for the positron-emitting species in the presence of a thousandfold excess carrier. Here, we describe the successful use of sensitive LC-MS/MS alone to identify and measure *t*_1/2_ and the SA of ^11^C-labeled tracers. The SA values obtained by this new method are compared with those from HPLC-radiometric methods. The LC-MS/MS method was found especially useful for verifying the validity of HPLC-radiometric methods.

## Methods

### Radiotracers

**Figure 1:**
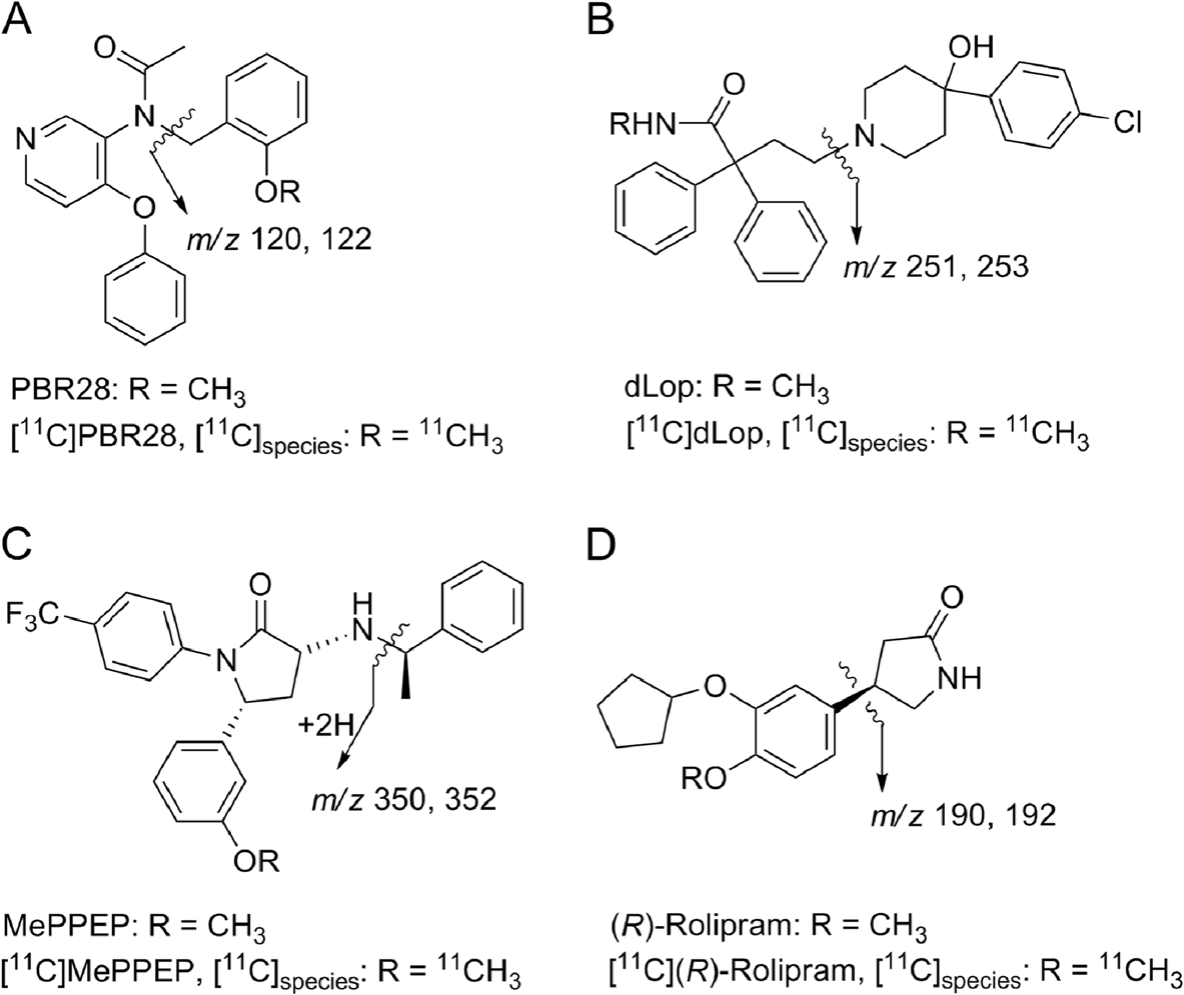
**Chemical structures of compounds and their **^**11**^**C-labeled analogs as PET tracers (A-D).** The bond cleavage site in generating product ion by collision-induced dissociation is shown for each molecule.

The PET radiotracers used were ^11^C]PBR28 (radioligand for translocator protein (18 kDa)) [16,17], ^11^C]dLop (radiotracer of *P*-glycoprotein function) [18,19], ^11^C]MePPEP (cannabinoid subtype 1 receptor radioligand) [20,21], and ^11^C](*R*)-rolipram (radioligand for cAMP phosphodiesterase IV) [22] (Figure 1A,B,C,D). Each radiotracer was prepared in high radiochemical purity (>99%) and formulated in sterile saline solution, as described previously [16-22].

### HPLC-radiometric measurement of SA

Freshly prepared and formulated radiotracer solution (100 μL), contained in a syringe, was measured for radioactivity in a calibrated ionization chamber (Atomlab 300, Biodex, Shirley, NY, USA) and then injected onto HPLC (Beckman, Fullerton, CA, USA). The radiotracer was eluted isocratically (acetonitrile(aq) 10 to 100 mM HCO_2_NH_4_ or 0.1% CF_3_CO_2_H) on a reverse-phase column (Onyx, Prodigy, or Luna, Phenomenex, Torrance, CA, USA) equipped with radioactivity and absorbance detectors. The mass of the carrier in the injectate was determined from a linear calibration curve generated from injections of known amounts of authentic non-radioactive standards. The SA was calculated from the decay-corrected activity present in the same volume of injectate, as measured in the calibrated ionization chamber.

### LC-MS/MS

Methods for radiotracer analyses were developed on an API 5000 LC-MS/MS system (AB Sciex, Foster City, CA, USA), consisting of Shimadzu LC (Columbia, MD, USA) interfaced via electrospray with triple quadrupole MS/MS operated in positive ionization mode. The technique principally involves quantitative measurement of two ions that differ by two mass-to-charge ratio (*m*/*z*) units, one for the ^11^C-labeled species, denoted [^11^C]_species_, and another for the carrier, denoted [M + 1]_carrier_. [M + 1]_carrier_ ions are generated from isotopologues having predominantly one carbon-13 atom or another heavy stable isotope (^2^H, ^15^N, or ^17^O) of very low abundance. Thus, for example, a solution of PBR28 was infused into the MS/MS, and, upon recording the product ions, compound-dependent and gas parameters were optimized for the *m*/*z* 349 [M + H]^+^ → 121 transition for monitoring [^12^C]_species_. An acquisition method for [^11^C]PBR28 was set up using the same instrument parameters and with the multiple-reaction monitoring table being edited to perform transitions, *m*/*z* 348 → 120 and *m*/*z* 350 → 122 for measuring [^11^C]_species_ and [M + 1]_carrier_, respectively (Figure 1A).

Similarly, MS/MS was tuned with dLop, MePPEP, and (*R*)-rolipram involving transitions *m*/*z* 463 [M + H]^+^ → 252, *m*/*z* 455 [M + H]^+^ → 351, and *m*/*z* 276 [M + H]^+^ → 191, respectively. Methods were set up to acquire product ions to measure [^11^C]_species_ and [M + 1]_carrier_ in these radiotracers. The dwell time in these methods ranged between 100 and 175 ms. In each method, the possible cross-talk between transitions was verified by inserting a dummy [M + H]^+^ → product ion transition between those for [^11^C]_species_ and [M + 1]_carrier_. The masses for the dummy transition differed by 5 to 10 amu from those for target ions. Each compound's product ion (Figure 1) was further verified by LC-MS^*n*^ (*n* = 2, 3) analysis in an ion-trap mass analyzer (LCQ Deca, Thermo Scientific, San Jose, CA, USA).

Each radiotracer sample was diluted four- to tenfold, and 2 to 5 μL of solution (<500 kBq) was injected with an autosampler (*n* = 6 or 12) at about 5-min intervals onto LC-MS/MS. Samples of [^11^C]PBR28, [^11^C]dLop, and [^11^C]MePPEP were chromatographed at 40°C on a C18 column (2 × 20 mm, 3 μm; Phenomenex) with a gradient of binary solvents (A/B; 400 μL/min), where A was 10 mM MeCO_2_NH_4_ in water/acetonitrile (90:10 *v*/*v*) and B was 10 mM MeCO_2_NH_4_ in acetonitrile/water (90:10 *v*/*v*). In the analysis of [^11^C]PBR28, for example, the pumps ran 70% A:30% B for 0.1 min and then a gradient reaching 20% A:80% B over 1 min. After 3.5 min, the mobile-phase composition was returned to the initial condition. [^11^C](*R*)-Rolipram was chromatographed on the same column eluted with a gradient of water (A) and acetonitrile (B) (A/B; 0.2% acetic acid): 80% A:20% B for 0.1 min, reaching 20% A:80% B over 2 min.

The relative abundance of the M + 1 peak was measured by injecting a solution of PBR28 onto the LC-MS/MS setup to perform *m*/*z* 349, 350 [M + H]^+^ → 121, 122 (^12^C, M + 1) transitions and thereby to provide *m*/*z* 122 to 121 peak area ratio (%). Other compounds were similarly analyzed using respective acquisition methods edited to monitor product ions of ^12^C and M + 1 species. The measured abundance (%) of M + 1 species relative to ^12^C was used for converting M + 1 into ^12^C peak area for radiotracer carrier. The peak area ratio (%) of M + 2 to M + 1 was measured by injecting 40 and 400 pg of PBR28 and acquiring transitions *m*/*z* 350, 351 → 122, 123.

### Calculations

The SA of the radiotracer (Bq/mol) was calculated as

(1) $$\frac{A_{M,M+1}^{*}}{A_{M+1,M}+A_{M,M+1}^{*}}\times {\text{SA}}_{\text{Theoretical}}$$

where (1) A^*^_M,M+1_ is the sum of the measured ^11^C, M]_species_ peak area and ^11^C, M + 1]_species_ area calculated from the relative abundances [23] of ^13^C, ^2^H, ^15^N, and ^17^O in the product ion; (2) *A*_M+1,M_ is the sum of the measured [M + 1]_carrier_ peak area and ^12^C]_carrier_ area calculated from the measured M + 1 abundance in the non-radioactive tracer; and (3) SA_Theoretical_ is the theoretical SA of carbon-11 (3.413 × 10^20^ Bq/mol), the product of ln2/*t*_1/2_ and Avogadro's number. The SA of the radiotracer was decay-corrected to give the SA at the end of radiosynthesis (SA_0_) as SAe^*λt*^, where *t* is the time between the end of synthesis and peak elution in LC-MS/MS and *λ* is 0.034 min^-1^ (ln2/20.38). A plot of lnSA (*n* = 12) versus clock time of peak elution in Prism 5.02 (GraphPad, La Jolla, CA, USA) gave the value for *λ* and thus *t*_1/2_ (*t*_1/2_ = ln2/*λ*).

## Results

In order to exemplify the non-radiometric LC-MS/MS method that we developed for identifying and measuring the SA of ^11^C-labeled tracers, we report results on four radiotracers applied in human PET studies, namely [^11^C]PBR28, [^11^C]dLop, [^11^C]MePPEP, and [^11^C](*R*)-rolipram (Figure 1). We illustrate the development of the procedure with the example of [^11^C]PBR28. In the MS/MS mode of a triple quadrupole analyzer, the [M + H]^+^ ion of non-radioactive PBR28 was dissociated and then one of the transitions, *m*/*z* 349 [M + H]^+^ → 121, suitable for monitoring [^11^C]_species_ and [M + 1]_carrier_ in [^11^C]PBR28 was selected, and instrument parameters were optimized. The MS/MS of PBR28 in an ion-trap analyzer also yielded the product ion, *m*/*z* 121, in high abundance. In the same mass analyzer, another dissociation (MS^3^) involving this target ion generated secondary product ions that were consistent with the genesis of the *m*/*z* 121 ion and thus the respective [^11^C]_species_ and [M + 1]_carrier_ ions by benzylic cleavage, as shown in Figure 1A.

LC-MS/MS of non-radioactive PBR28 showed transitions *m*/*z* 349 [M + H]^+^ → 121 (^12^C) and 350 [M + H]^+^ → 122 (M + 1) associated with a peak eluting at 1.61 min on a short reverse-phase LC column. The measured relative abundance (8.88%) of the *m*/*z* = 122 for M + 1 species was close to the value (8.83%) calculated for the product ion formed by the benzylic cleavage of the [M + H]^+^ ion of PBR28 (Figure 1A). Subsequently, the MS/MS acquisition method was edited to monitor the *m*/*z* 348 → 120 transition for [^11^C]_species_ in [^11^C]PBR28. As expected, a similar LC-MS/MS analysis of non-radioactive PBR28 showed no peak for an *m*/*z* 348 → 120 transition. Furthermore, analysis of a fully decayed preparation of [^11^C]PBR28 showed no peak for this transition. Thus, carrier PBR28 did not generate a peak due to the *m*/*z* 120 ion or any other spurious peak when performing the *m*/*z* 348 → 120 transition. Moreover, a dummy transition, entered between the transitions for [^11^C]_species_ and [M + 1]_carrier_, showed no cross-talk during the acquisition.

The possibility that coeluting carrier PBR28 might affect the ionization of [^11^C]PBR28 was tested by injecting high (400 pg) and low (40 pg) amounts of PBR28 into the LC-MS/MS and acquiring the product ions from [M + H]^+^ of the M + 1 and M + 2 species. The peak area ratio (%; mean ± standard deviation (SD); *n* = 3) of M + 2 to M + 1 was 6.64 ± 0.02 at 400 pg and 6.52 ± 0.09 at 40 pg of PBR28. These ratios agreed well with the calculated M + 2 to M + 1 ratio of 6.56%. Thus, excess [^12^C]_carrier_ did not cause either [^11^C]_species_ or [M + 1]_carrier_ to ionize differently, and their ion currents were therefore expected to represent their respective concentrations.

**Figure 2:**
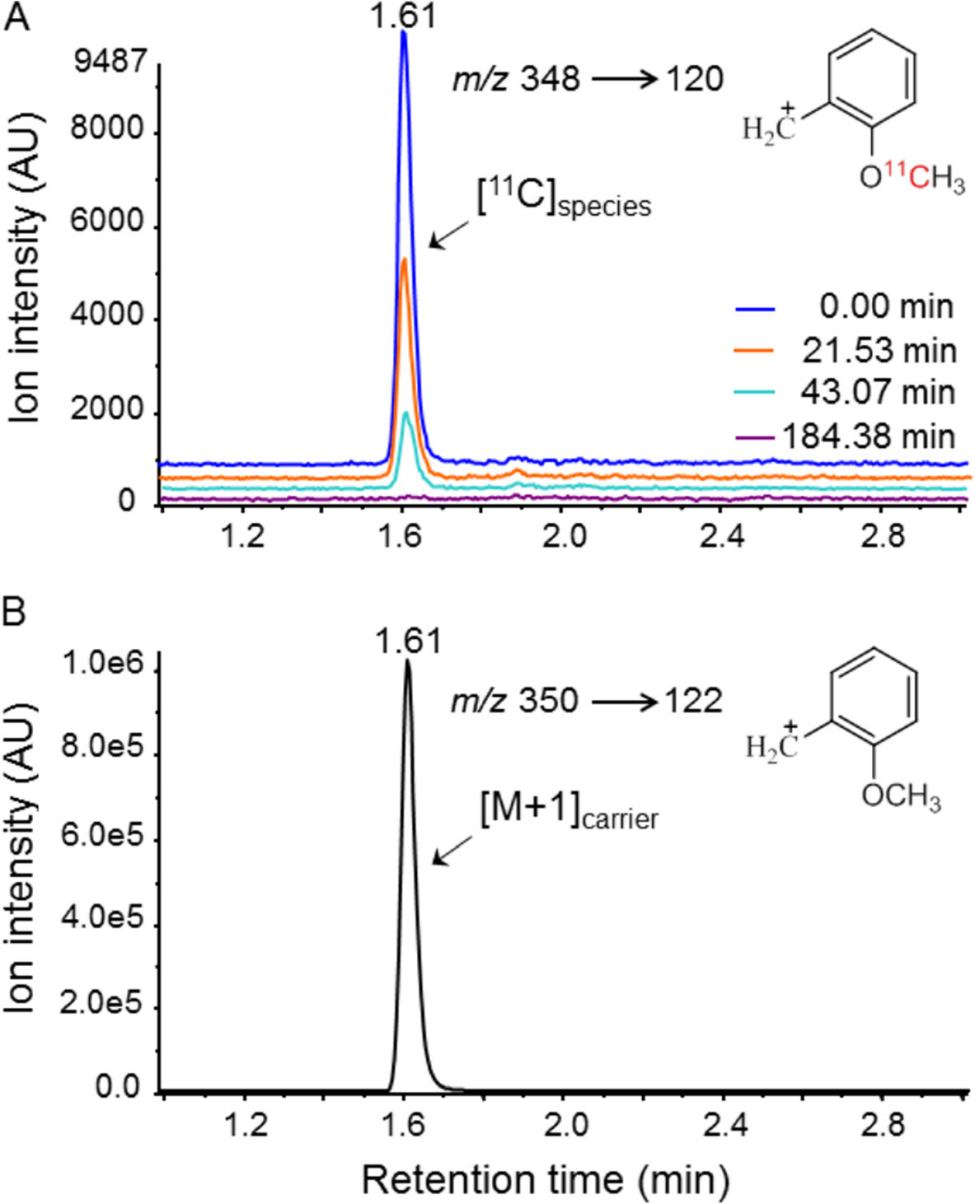
**LC-MS/MS of the same [**^**11**^**C]PBR28 preparation at four time-points of carbon-11 decay.** The ion chromatogram overlay showing peaks for [^11^C]_species_ as it decayed (**A**) and unchanging peak for [M + 1]_carrier_ (**B**) during the same analysis. Their respective product ions *m*/*z* 120 and 122 were monitored.

MS/MS of a sample of freshly prepared [^11^C]PBR28 (approximately 400 kBq) detected an *m*/*z* 348 → 120 transition specific for the [^11^C]_species_ and *m*/*z* 350 → 122 for the constituent [M + 1]_carrier_. The LC peaks (Figure 2A,B) due to the product ions, *m*/*z* 120 and 122 for [^11^C]_species_ and [M + 1]_carrier_, respectively, appeared in the ion chromatogram at the same *t*_R_ as reference PBR28. As shown with ion chromatogram overlays, the peak for the [^11^C]_species_ with *m*/*z* = 120 diminished in intensity and was undetectable after elapse of about six *t*_1/2_ of carbon-11. The intensity of the peak for [M + 1]_carrier_ remained constant throughout the analysis, as expected. Mass detection by MS/MS of [^11^C]_species_ along with [M + 1]_carrier_ as LC peaks at the *t*_R_ of PBR28 confirmed the identity of the [^11^C]_species_ including the position of the carbon-11 atom as being in the methoxy group of the methoxybenzyl moiety (Figure 1A).

LC-MS/MS methods were similarly set up for the characterization of [^11^C]_species_ and [M + 1]_carrier_ in [^11^C]dLop, [^11^C]MePPEP, and [^11^C](*R*)-rolipram preparations. Analysis of [^11^C]dLop showed LC peaks at *t*_R_ = 2.02 min due to the product ions *m*/*z* 251 and 253 of [^11^C]_species_ and [M + 1]_carrier_, respectively (Figure 1). [^11^C]MePPEP showed LC peaks at *t*_R_ = 2.19 min arising from the acquisition of product ions *m*/*z* 350 and 352 of [^11^C]_species_ and [M + 1]_carrier_, respectively. Similarly, analysis of [^11^C](*R*)-rolipram showed LC peaks at *t*_R_ = 1.96 min upon acquiring the product ions *m*/*z* 190 and 192 of [^11^C]_species_ and [M + 1]_carrier_, respectively.

The LC-MS/MS measurements of [^11^C]_species_ and carrier gave the mole fraction of the [^11^C]_species_, which, upon multiplication with the theoretical SA of carbon-11, gave the SA of the radiotracer (Equation 1). In calculating the mole fraction of [^11^C]_species_, the measured peak area for [M + 1]_carrier_ was converted into that for [^12^C]_carrier_ using the previously measured [M + 1]/^12^C ratio in PBR28, dLop, MePPEP, or (*R*)-rolipram. This indirect measurement had the benefit of neatly circumventing possible saturation of the detector by [^12^C]_carrier_ at the concentration of the radiotracer needed for measuring [^11^C]_species_. Also, in this method, the masses of acquired ions differed by two units, and any contribution of [^11^C, M + 2]_species_ to the [M + 1]_carrier_ abundance would therefore have been negligible.

As examples, the measured peak areas of [^11^C]_species_ and [M + 1]_carrier_ and the derived peak areas used for calculating SA are shown for [^11^C]PBR28 and [^11^C](*R*)-rolipram in Table 1. These data show that the peak area and area ratio for [^11^C]_species_ and the SA were halved after elapse of one *t*_1/2_ of carbon-11 while the peak area for [M + 1]_carrier_ remained constant, as expected. The measurement of [^11^C]_species_ and [M + 1]_carrier_ is quantitative as reflected in the decay-corrected SA values. The area ratio 7.9240 × 10^−4^ for [^11^C]PBR28 implies that 1 out of 1,262 molecules was labeled with carbon-11 at the time of MS/MS detection. This ratio is consistent with the low degree of radionuclide enrichment found in ^11^C-labeled preparations. Also, in Table 1, the peak areas for [^11^C](*R*)-rolipram are for a preparation injected onto LC-MS/MS after elapse of 30.88 and 52.38 min from the end of synthesis. The comparable decay-corrected SA values indicate good accuracy of the measurement even after elapse of two *t*_1/2_ of the radiotracer.

**Table 1:** **Calculation of specific radioactivity from the peak areas of [**^**11**^**C]**_**species **_**and [M + 1]**_**carrier **_**in radiotracers**

**Radiotracer**	**Elution time**^**a**^	**Peak areas**					**SA**^**e**^**(GBq/μmol)**	**SA**^**f**^**(GBq/μmol)**
		^**11**^**C**	^**11**^**C**	**M + 1**	^**12**^**C**	**Ratio**		
	**(h:min:s)**	**×10**^**−4**^	**×10**^**−3b**^	**×10**^**−6**^	**×10**^**−7c**^	**×10**^**4d**^		
[^11^C]PBR28	10:21:23	2.3920	1.8531	2.6506	2.9849	7.9240	270.4	330.3
	10:42:55	1.2103	0.9376	2.7072	3.0486	3.9271	134.0	340.6
[^11^C](*R*)-Rolipram	11:15:49	0.5745	0.7009	1.5974	1.1970	4.7490	162.1	463.3
	11:37:19	0.2827	0.3449	1.6280	1.2199	2.2936	78.3	464.9

^a^Elution time for coeluting LC peaks of [^11^C]_species_ and [M + 1]_carrier_; ^b^calculated peak area for [^11^C, M + 1]_species_ (Equation 1); ^c^calculated peak area from measured M + 1/^12^C ratio in PBR28 and (*R*)-rolipram; ^d^peak area ratio of [^11^C]_species_ to the sum of carrier and [^11^C]_species_; ^e^specific radioactivity calculated from the peak area ratio; ^f^decay-corrected specific radioactivity.

**Figure 3:**
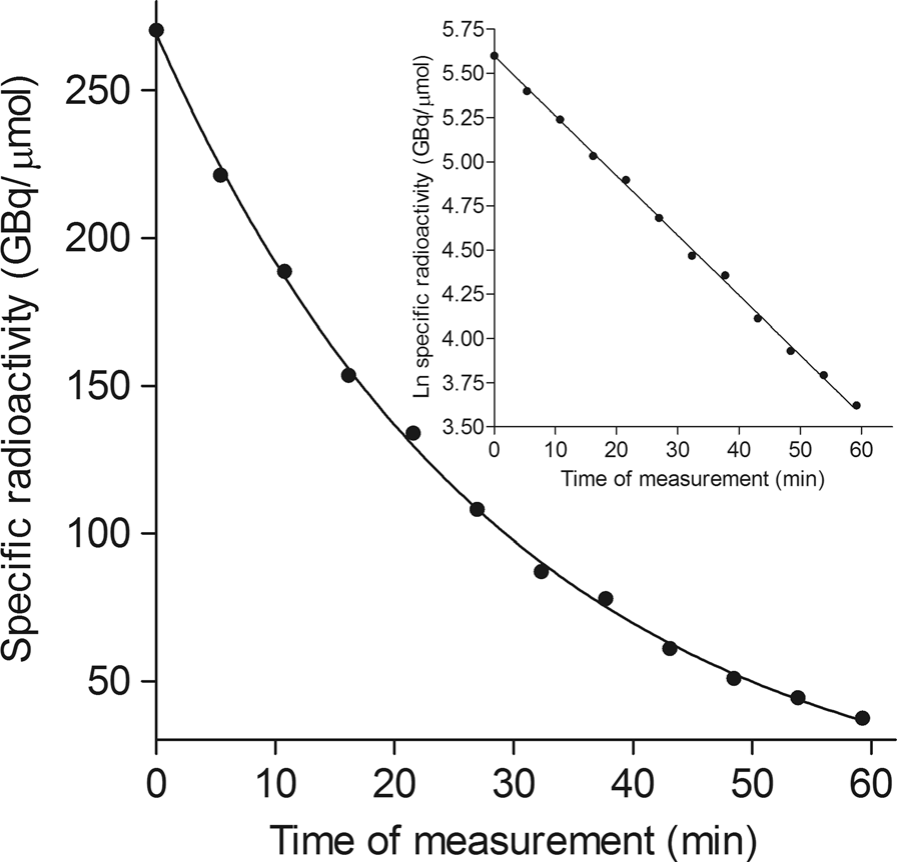
**Plot of specific radioactivity of [**^**11**^**C]PBR28 versus time.** It shows exponential loss of [^11^C]_species_ mass due to radionuclide decay. A semilog plot of the same data is shown in the inset.

The accuracy of the LC-MS/MS method for measuring the SA of PET radiotracers was further supported through monitoring the loss of mass of ^11^C]_species_ as the radionuclide decayed. Three preparations each of ^11^C]PBR28 and ^11^C](*R*)-rolipram and two of ^11^C]dLop were analyzed by injecting each 12 times at known intervals of about 5 min. The SA of the radiotracer was calculated for each injection at the clock time of the peak elution in the ion chromatogram. Plots of the natural logarithm of SA versus measurement time gave straight lines (*r*^*2*^ > 0.99) as shown, for example, for a ^11^C]PBR28 preparation (Figure 3). In each case, the decay constant *λ* for carbon-11 was obtained from the negative slope of the regression equation (*y* = −*λx* + *b*). The *t*_1/2_ (*t*_1/2_ = ln2/*λ*) of carbon-11 was estimated from these decay constants to be 20.43 ± 0.24 min (mean ± SD; *n* = 8), which is in excellent agreement with the accepted value of 20.38 min ascertained by measuring *β*^+^ emission [24]. The accuracy of these *t*_1/2_ measurements with LC-MS/MS implies that the measured peak areas of decaying ^11^C]_species_ and the stable [M + 1]_carrier_ truly represent their concentrations.

Decay-corrected SA was measured with the LC-MS/MS method on three preparations of each of the four radiotracers (Table 2). Each preparation was measured six times over a period of 30 min. These measurements were remarkably reproducible (<5% RSD). Before LC-MS/MS analysis, each radiotracer was also analyzed by the calibrated radiometric method (activity using an ionization chamber dose calibrator and mass using HPLC apparatus) regularly used in our laboratory for quality control. Each analysis required at least 10 min for completion and was performed only once because of the time constraint imposed by the short half-life. The SA measurements obtained with LC-MS/MS deviated by ±7.4% overall from the mean of both radiometric and LC-MS/MS methods (Table 2). The possible sources of error in the radiometric method include both the HPLC apparatus and the dose calibrator used for measuring carrier and radioactivity, respectively. The HPLC method used a linear calibration curve (*r*^*2*^ > 0.99) generated with authentic non-radioactive compounds, such as, for example, PBR28 (>99% pure; RTI International, Research Triangle Park, NC, USA) or (*R*)-rolipram (>98%; Tocris, Ellisville, MO, USA). The quantification of the carrier may not always be accurate due to lack of an internal standard and possible presence of a coeluting impurity. Measurements of the radioactivity of ^11^C-labeled preparations (*n* = 6) in one ionization chamber were compared with measurements in another to assess their reliability. The two ionization chambers showed 10% to 12% differences in the radioactivity measurements even though both detectors had been identically calibrated. The lack of accuracy and reproducibility in measurements with ionization chambers [10-14] may at least partially explain the difference in SA measured by radiometric-HPLC and LC-MS/MS methods. Other radiochemical detectors likely suffer similar deficiencies, especially as surrogate isotopes are also used for their calibration.

**Table 2:** Specific radioactivity (decay-corrected) of PET radiotracers measured by LC-MS/MS and radiometric methods

**Radiotracer**	**SA (GBq/μmol)**		**Difference**^**b**^**(%)**
	**LC-MS/MS**^**a**^	**Radiometry**	
[^11^C]PBR28	265.5 ± 8.5	290.8	−9.1
	330.4 ± 5.8	363.6	−9.6
	167.0 ± 2.9	173.3	−3.7
[^11^C]dLop	234.1 ± 3.2	237.9	−1.6
	227.1 ± 6.1	253.2	−10.9
	241.4 ± 7.0	264.0	−9.1
[^11^C]MePPEP	160.1 ± 7.2	151.5	+5.5
	244.0 ± 11.7	238.0	+2.5
	82.5 ± 3.0	92.4	−11.3
[^11^C](*R*)-Rolipram	473.9 ± 4.8	459.9	+3.0
	505.0 ± 9.5	450.5	+11.4
	459.0 ± 5.8	411.1	+11.0

^a^Mean ± SD specific radioactivity from six consecutive analyses; ^b^Difference (%) = [(LC-MS/MS − radiometry) / mean of both methods] × 100.

## Discussion

Here, we demonstrated the capability of electrospray ionization (ESI)-MS/MS to ionize and isolate [M + H]^+^ of [^11^C]_species_ and then generate and detect the product ion-bearing carbon-11. The mass-specific detection of [^11^C]_species_ along with [M + 1]_carrier_ at the expected *t*_R_ allowed unambiguous identification of the radiotracer. By monitoring [M + 1]_carrier_, instead of the carbon-12 counterpart, we avoided saturation of the MS/MS detector during simultaneous measurement of trace concentrations of [^11^C]_species_. The measurement of both species gave the SA and *t*_1/2_ of the radiotracer while also confirming radiotracer identity. The results from multiple analyses showed that the LC-MS/MS method is fast, accurate, and reproducible enough for characterizing low-enriched, fast-decaying radiotracers. This mass-specific detection method is suitable for validating the conventional method of characterization of PET radiotracers.

We verified that the excess carrier does not impact the ionization and detection of coeluting [^11^C]_species_ in our LC-MS/MS system. The peak for [^11^C]_species_ declined at an expected rate and then disappeared from the ion chromatogram as carbon-11 in the product ion decayed. Secondly, the ratio of M + 2 to M + 1 isotopologues was not altered when the radiotracer's non-labeled standard was injected at a concentration comparable to that of the carrier in the radiotracer. Also, the concentrations of [^11^C]_species_ and [M + 1]_carrier_ in radiotracers were within the dynamic range of detection of the triple quadrupole MS/MS system. Before being injected into LC-MS/MS, the radiotracer preparations were diluted adequately to avoid possible saturation of the ionization and detection systems. Furthermore, no cross-talk was observed between the [M + H]^+^ → product ion transition for [^11^C]_species_ and that for [M + 1]_carrier._

Carbon-11 decays to a stable nuclide, boron-11. This energetic decay event would instantly transform any molecule in which the carbon-11 had been incorporated. The product of carbon-11 decay would have a *t*_R_ and mass different from those of the [^11^C]_species_ or the [M + 1]_carrier_. Accordingly, the LC-MS/MS acquisition detected no product or chemical impurity from the decay of any of these PET radiotracers. The measurement showed exponential loss of mass of [^11^C]_species_ as the radionuclide decayed thereby gave the same expected *t*_1/2_ value for each radiotracer.

The radiometric method of SA measurement involves two detection systems, and both are prone to errors. For example, we observed a significant difference between two dose calibrators in recording the radioactivity of the same radiotracer preparation. The sources of error in the HPLC measurement of mass may include the weighing of standard, preparation of the calibration curve solutions, injection, chromatography, and UV detection. In contrast, the LC-MS/MS technique measures the mole fraction of [^11^C]_species_ in a radiotracer similar to the isotope ratio in a compound. Thus, SA was obtained simply by entering into a spreadsheet: (1) the measured LC-MS/MS peak areas of [^11^C]_species_ and [M + 1]_carrier_, (2) the abundance of [M + 1]_carrier_ measured in the reference standard, (3) the area for the [^11^C, M + 1]_species_ ion calculated from the natural abundance of heavy stable isotopes in [^11^C]_species_, and (4) the theoretical maximum SA of a compound labeled with one carbon-11 atom, as detailed under the ‘Calculations’ subsection.

The rapid LC-MS/MS method described here enables definitive characterization of radiotracers used in human PET imaging. A mass-specific detection method such as this is not susceptible to errors commonly associated with the use of the radio-HPLC method, as discussed. It may not be feasible to use triple quadrupole MS/MS regularly; however, it provides a wholly independent non-radiometric means for confirming identity and verifying SA of a radiotracer when needed. A less expensive and more commonly used single quadrupole MS instrument may offer the sensitivity to measure ^11^C]_species_ and [M + 1]_carrier_ by selected ion monitoring of respective [M + H]^+^ following ESI. However, such a less specific MS method can work only if no [M − 1]^+^ ion is generated during ionization. Other mass analyzers such as time-of-flight and linear ion traps may also be suitable for the characterization of PET radiotracers. Finally, the LC-MS/MS method should be applicable to all ^11^C-labeled tracers, except in instances where no ^11^C-containing product ion is obtained or where interfering ions are generated. If a labeled product ion is not available, selected ion monitoring of a precursor ion ([M + H]^+^) with a high-resolution MS can substitute the MS/MS method for measuring ^11^C]_species_ and [M + 1]_carrier_. Also, a high-resolution MS is likely to resolve any interfering ions of isobaric species from the product ion of ^11^C]_species_ in ^11^C-labeled tracers. In principle, LC-MS/MS may be used for characterizing PET radiotracers labeled with other short-lived positron emitters. Although an isotope separator has been used to identify the position of ^18^F in labeled 1,1,1,2-tetrafluoroethane [25], no parallel mass spectrometric method has yet been reported for measuring the SA of ^18^F-labeled tracers. The LC-MS/MS method should also be suitable for this purpose, as shown with the analysis of an ^18^F-labeled tracer in our preliminary report [26].

## Conclusions

A simultaneous mass detection of [^11^C]_species_ and [M + 1]_carrier_ in ^11^C-labeled PET tracers was achieved using the LC-MS/MS technique. A single rapid analysis can verify the identity including the position of label in a ^11^C-labeled tracer preparation as well as provide a fully independent measure of SA. The LC-MS/MS method is a valuable adjunct to other techniques used in the quality control of PET radiopharmaceuticals.

## Abbreviations

[^11^C]_species_: carbon-11 species; [M + 1]_carrier_: ^13^C, ^2^H, ^15^N, or ^17^O carrier isotopologue; ESI: electrospray ionization; HPLC: high-performance liquid chromatography; LC-MS/MS: liquid chromatography-mass spectrometry/mass spectrometry; PET: positron-emission tomography; SA: specific radioactivity; *t*_R_: HPLC or LC retention time.

## Competing interests

The authors declare that they have no competing interests.

## Authors’ contributions

HUS and VWP designed the experiments and wrote the manuscript. HUS and CLM performed the LC-MS/MS and processed the data. CLM and YZ prepared the radiotracers and performed the HPLC measurements. HUS conceived the study and interpreted the data. All authors read and approved the final manuscript.
